# Topical Pain Management: An Updated Review of Current Evidence and Emerging Strategies

**DOI:** 10.3390/jcm15135311

**Published:** 2026-07-07

**Authors:** Urszula Adamiak-Giera, Patryk Rzeczycki, Magdalena Sawczuk, Oliwia Pęciak, Monika Białecka

**Affiliations:** 1 Department of Pharmacokinetics and Therapeutic Drug Monitoring, Pomeranian Medical University in Szczecin, 70-111 Szczecin, Poland; peciak.oliwia@gmail.com (O.P.); monika.bialecka@pum.edu.pl (M.B.); 2 Department of Experimental and Clinical Pharmacology, Pomeranian Medical University in Szczecin, 70-111 Szczecin, Poland; patryk.rzeczycki@pum.edu.pl; 3 Laboratory of Pharmacodynamics, Pomeranian Medical University in Szczecin, 71-899 Szczecin, Poland; magdalena.sawczuk@pum.edu.pl

**Keywords:** chronic pain, topical analgesia, topical therapy, neuropathic pain, nociceptive pain, nociplastic pain, NSAIDs, lidocaine, capsaicin, topical NSAIDs, pain management, osteoarthritis, postherpetic neuralgia, peripheral neuropathy, multimodal analgesia, transdermal drug delivery, local anesthetics, neuroinflammation, central sensitization, topical pharmacotherapy

## Abstract

**Introduction**: Pain is one of the most common reasons why patients seek medical care, and chronic pain is now recognized as a major health problem worldwide. Better understanding of pain mechanisms has shown the importance of distinguishing nociceptive, neuropathic, and nociplastic pain in order to choose the most effective treatment. In recent years, topical analgesics have gained increasing attention because they can provide pain relief directly at the site of application while reducing systemic exposure and the risk of adverse effects. This is especially important in older adults, patients with multiple diseases, and those exposed to polypharmacy. **Methods**: This narrative review presents the current knowledge on the pharmacology, efficacy, and safety of topical drugs used in pain treatment. Particular attention is given to topical non-steroidal anti-inflammatory drugs (NSAIDs), lidocaine, capsaicin, menthol, and camphor. The review also discusses newer and less established therapies used mainly in neuropathic pain, including topical ketamine, amitriptyline, phenytoin, gabapentin, and clonidine. A structured, non-systematic literature search was conducted using the PubMed/MEDLINE, Scopus, Web of Science, and Google Scholar databases to identify studies evaluating the efficacy and safety of topical analgesic therapies. **Results**: Current evidence supports topical NSAIDs as first-line therapy for localized musculoskeletal pain and osteoarthritis, while lidocaine and high-concentration capsaicin patches are effective options in focal neuropathic pain. Although several newer topical therapies show promising results, more high-quality clinical studies are still needed. Overall, topical analgesia is an important part of multimodal pain management because it combines analgesic efficacy with a better safety profile compared with many systemic therapies. **Conclusions**: Taking the aspects discussed in this paper into account, it seems justified to search for new drug combinations that would contribute to effective pain therapy with topical agents. It is recognized that a multimodal approach to pain management, which utilizes drugs with different mechanisms of action, can increase efficacy and reduce the systemic adverse events of the drugs used. The effective and safe treatment of patients with pain, especially neuropathic pain, despite emerging new clinical trials, remains a challenge for clinicians.

## 1. Introduction

Pain is the most common symptom among patients seeking medical help, especially in primary health care and emergency departments [1]. The modern definition of pain, adopted by the International Association for the Study of Pain (IASP), emphasizes that pain is both a sensory and emotional experience associated with actual or potential tissue damage [2]. In physiology, pain serves a warning and protective function: it is a signal that triggers a reflex and behavioral response from the body to limit the consequences of damage (e.g., offloading, stimulus avoidance, increased vigilance) [3]. However, the above definition primarily refers to acute pain, whereas chronic pain can be classified as a major health problem in its own right. The World Health Organization (WHO) and the International Association for the Study of Pain (IASP) (e.g., in the context of the ICD-11 classification) increasingly treat chronic pain not merely as a “symptom,” but as a distinct disease entity requiring diagnosis and treatment, particularly when it is the patient’s primary clinical problem [4]. The scale of the burden is enormous; the IASP estimates that ~20% of the global population lives with chronic pain [5].

The mechanisms underlying the chronification of acute pain are highly complex and include, among others, neuroinflammatory activation of the nervous system, ion channel dysregulation, and the perpetuation of peripheral and central sensitization [6]. It should be emphasized that one of the causes of the development of chronic pain is the inadequate treatment of acute pain [7].

This narrative review was conducted to summarize the current evidence on the efficacy, safety, and practical use of topical agents in the treatment of chronic pain. Particular attention was paid to well-established therapies, such as topical non-steroidal anti-inflammatory drugs (NSAIDs), lidocaine, and capsaicin, as well as newer and emerging treatments used mainly in neuropathic pain.

A structured, non-systematic literature search was performed using the PubMed/MEDLINE, Scopus, Web of Science, and Google Scholar databases. Publications published between 1991 and 2026 were considered, with a primary focus on studies published between 2004 and 2026 that evaluated the clinical effectiveness of topical analgesic therapies. Review articles, meta-analyses, randomized clinical trials, observational studies, and original experimental studies were included in the analysis.

The search strategy used the following keywords and their combinations: *chronic pain*; *topical analgesia*; *topical therapy*; *neuropathic pain*; *nociceptive pain*; *nociplastic pain*; *NSAIDs*; *lidocaine*; *capsaicin*; *topical NSAIDs*; *pain management*; *osteoarthritis*; *postherpetic neuralgia*; *peripheral neuropathy*; *multimodal analgesia*; *transdermal drug delivery*; *local anesthetics*; *neuroinflammation*; *central sensitization*; *topical pharmacotherapy*. Boolean operators (“AND” and “OR”) were used to refine the search.

Only full-text articles published in English were included. The selected publications addressed the pharmacology, mechanisms of action, clinical efficacy, safety, and practical applications of topical analgesics. Particular emphasis was placed on evidence from randomized controlled trials, systematic reviews, meta-analyses, and current clinical guidelines.

This review was not designed as a systematic review or meta-analysis. Its aim was to provide clinicians with a practical overview of the currently available evidence on topical pain therapies, discuss their mechanisms of action and clinical applications, and identify areas where further research is needed.

## 2. Methods

This narrative review presents the current knowledge on the pharmacology, efficacy, and safety of topical drugs used in pain treatment. Particular attention is given to topical non-steroidal anti-inflammatory drugs (NSAIDs), lidocaine, capsaicin, menthol, and camphor. The review also discusses newer and less established therapies used mainly in neuropathic pain, including topical ketamine, amitriptyline, phenytoin, gabapentin, and clonidine. A structured, non-systematic literature search was conducted using the PubMed/MEDLINE, Scopus, Web of Science, and Google Scholar databases to identify studies evaluating the efficacy and safety of topical analgesic therapies.

## 3. Recognizing the Pain Mechanism as the Basis for Selecting Appropriate Treatment

Establishing a correct diagnosis is the foundation of treatment; therefore, new drugs and/or routes of administration that increase the efficacy of pain pharmacotherapy are constantly being sought. The International Association for the Study of Pain defines nociceptive pain as pain arising from the activation of nociceptors in response to actual or threatened damage to non-neural tissue [8]. Neuropathic pain is pain caused by a lesion or disease of the somatosensory nervous system [9]. The third category—nociplastic pain—is fundamentally characterized by the dysfunction of signal processing in the central nervous system, which consists of the amplification of neuronal responses in the spinal cord (allodynia, hyperalgesia); the failure of pain inhibition systems combined with the facilitation of its conduction; functional changes in the brain; and microglial activation, leading to the overinterpretation of stimuli as threatening [10]. It encompasses situations where pain arises from “altered nociception,” and the clinical picture is not fully explained by either a nociceptive (tissue) or neuropathic (somatosensory system damage) mechanism. In practice, nociplastic pain co-occurs with sleep disturbances, fatigue, or cognitive problems [11]. Due to the possible co-occurrence of different types of pain, it is currently accepted when defining the type of ailment to use the term: pain with a predominant nociceptive, neuropathic, or nociplastic component [12].

The mechanisms underlying the three described types of pain may be similar due to the shared involvement of functional disturbances at the level of ion channels, neurotransmitters, and inflammatory mediators:

- In inflammatory pain, prostaglandins (e.g., PGE2) increase nociceptor excitability, inter alia, via EP receptors (prostaglandin E receptor) and secondary pathways (cAMP/PKA, PKC) (cyclic adenosine monophosphate/protein kinase A, protein kinase C), which can be associated with a lowered activation threshold for pain receptors and ion channels; classically, this explains why inhibiting COX-2 (cyclooxygenase-2) and reducing prostaglandin synthesis produces an analgesic effect [13].
- In neuropathic pain, ectopic discharges and hyperexcitability of sensory fibers are observed, where sodium channels (e.g., NaV) and sensitization phenomena play a significant role [14].
- In chronic pain, the neuroinflammatory and glial component (microglia, astrocytes) is perpetuated, which amplifies pain transmission and promotes central sensitization [15].

In clinical practice, this means that:

- Nociceptive pain (e.g., soft tissue injury, periarthritis, osteoarthritis pain) can often be effectively treated with anti-inflammatory drugs, including topical NSAIDs, because the target is the COX–prostaglandin cascade [16].
- Neuropathic pain more often requires drugs that modulate nerve excitability (e.g., lidocaine) or the desensitization of nociceptor endings (high-concentration capsaicin); however, the efficacy of systemic pharmacotherapy is sometimes moderate, and tolerance is limited [17].
- Nociplastic pain is difficult to treat without adhering to specific recommendations. Cyclobenzaprine hydrochloride, selective serotonin and norepinephrine reuptake inhibitors (SNRIs), and the treatment of co-occurring symptoms are among the treatments of choice. Anti-inflammatory drugs used in monotherapy are ineffective, and opioid analgesics are not recommended [18].

**Figure 1:**
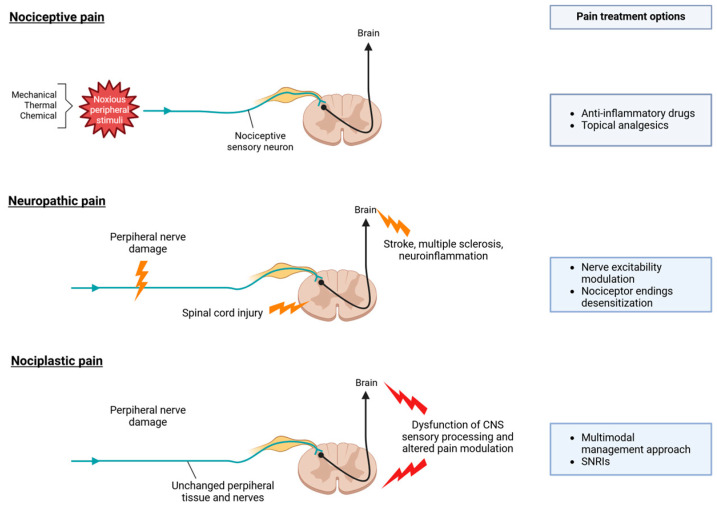
Choosing an appropriate approach to pain management depends on the pain type. CNS—central nervous system; SNRis—serotonin and norepinephrine reuptake inhibitors.

Figure 1 presents the identification of the pain mechanism as the basis for selecting appropriate treatment.

Currently, the rationale/popularity of using topical preparations is increasingly pointed out because local treatment is characterized by a better safety profile compared to systemic treatment, especially in older adults, patients with multimorbidity, or those subjected to polypharmacy [19]. A primary principle of pain pharmacotherapy is also the combination of drugs with different mechanisms of action in order to increase the analgesic effect while simultaneously reducing the risk of adverse events. This principle also applies to topically administered drugs [20] (Table 1).

## 4. Topical Pharmacology: Skin Barrier and Drug Distribution

In addition to analgesic efficacy and the adverse event profile, it is increasingly pointed out that the route of drug administration plays a key role in achieving effective analgesic therapy and minimizing complications [24]. The route of administration should be appropriately selected, taking into account the causes of pain, the patient’s clinical condition, comorbidities, and age [25]. Within the described methods of drug application, the following are distinguished:

- Topical (local) drug application—acting primarily at the site of application (skin, superficial tissues, joint area) [26].
- Transdermal (percutaneous) application—aimed at achieving a systemic effect, i.e., the penetration of the drug into the systemic circulation [27].

After the application of a topical drug, it must penetrate the stratum corneum of the epidermis, which is the primary diffusion barrier [28]. The classic “500 Dalton rule” states that molecules with a mass > 500 Da have great difficulty penetrating intact skin, which in practice limits the number of drugs that can be used in this form to achieve adequate local treatment efficacy as well as an appropriate drug concentration in the blood and biophase [29]. Therefore, substances with a relatively low molecular weight are frequently used in topical analgesia. It is also important to consider the nature of the substance (lipophilic vs. hydrophilic) in order to effectively overcome the lipid barrier of the stratum corneum and reach the aqueous environment of the tissues, as well as the type of formulation that will ensure the optimal drug release (gel, ointment, patch, solution, microemulsion, etc.) [30].

## 5. Efficacy and Safety of Topical Drugs

**Figure 2:**
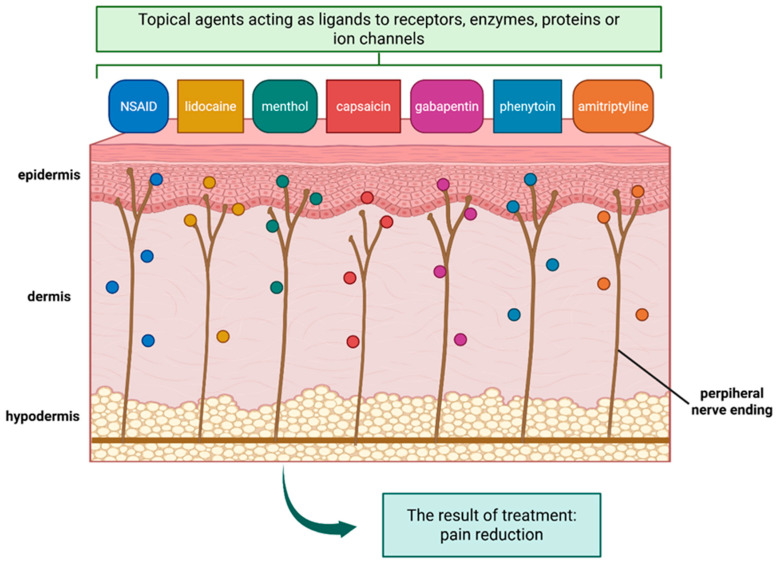
Topical application of pain-relieving drugs.

For topical NSAIDs and lidocaine, the answer is often “yes,” which can be observed in their pharmacokinetics; for example, 1% diclofenac gel provides a systemic exposure 5–17 times lower than the oral formulation, while simultaneously maintaining effective local action [31]. From a regulatory perspective, it is also increasingly evident that a topical preparation is a “complex” product [32]. The U.S. Food and Drug Administration (FDA) is developing bioequivalence standards for topical products based on in vitro release and permeation testing (IVRT/IVPT), because the classic “blood concentration” does not always reflect the true efficacy of a topical preparation [33] (Table 2 and Figure 2).

The choice of a specific topical preparation depends on the predominant pain mechanism:

- Non-steroidal anti-inflammatory drugs (NSAIDs) are the gold standard in nociceptive pain (e.g., injuries, osteoarthritis), e.g.: diclofenac, etofenamate, ibuprofen, naproxen, ketoprofen [38].
- Local anesthetics are used primarily in localized neuropathic pain (e.g., postherpetic neuralgia), e.g.: lidocaine (most commonly in the form of 5% patches) [39].
- TRPV1 receptor agonists are utilized in the therapy of persistent neuropathic pain, e.g.: capsaicin (low concentrations in ointments or high 8% concentration in patches) [40].

## 6. Non-Steroidal Anti-Inflammatory Drugs (NSAIDs)

**Figure 3:**
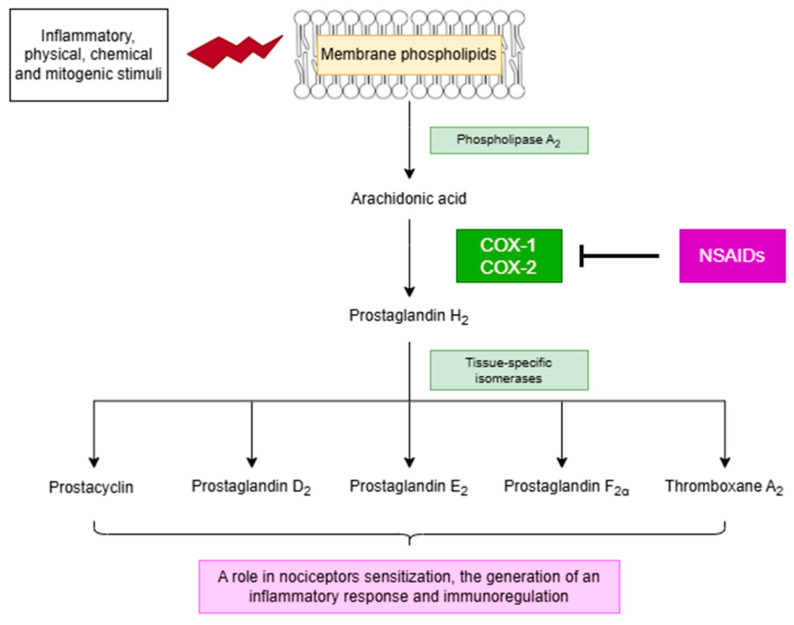
Mechanism of action of NSAIDs. COX-1, COX-2—cyklooksygenase 1,2; NSAIDs—non-steroidal anti-inflammatory drugs.

Despite immense progress in pain pharmacotherapy and the introduction of new drugs to the pharmaceutical market, NSAIDs constitute a class of drugs that remains widely used in the broadly understood pain management strategy. Topically applied NSAIDs can be used on intact skin in the form of creams, ointments, gels, sprays, and patches; however, the specific drug formulations differ in their skin penetration and, consequently, the degree of active substance absorption [41,42]. Following topical application, drugs from this class are absorbed, reaching therapeutic concentrations in the surrounding tissues, whereas the serum concentration of the topically administered drug is significantly lower compared to oral administration [43,44]. Thus, in contrast to systemic administration, the topical application of NSAIDs allows for achieving an analgesic and anti-inflammatory effect with a significantly lower risk of adverse events [C] [45]. Figure 3 presents the mechanism of action of non-steroidal anti-inflammatory drugs (NSAIDs).

Chronic pain is a major problem for individuals with osteoarthritis (OA). Although there is a lack of approved disease-modifying therapies, multiple organizations such as the Osteoarthritis Research Society International (OARSI), American College of Rheumatology (ACR), American Academy of Orthopaedic Surgeons (AAOS), European Alliance of Associations for Rheumatology (EULAR), and the European Society for Clinical and Economic Aspects of Osteoporosis, Osteoarthritis and Musculoskeletal Diseases (ESCEO) recommend the topical use of NSAIDs as first-line drugs due to the lower risk of systemic adverse events, especially during chronic therapy [D] [46,47,48]. These recommendations primarily concern knee osteoarthritis, and conditionally polyarticular osteoarthritis without comorbidities [21,49]. Similarly, the ACR recommends the topical use of NSAIDs in osteoarthritis of the knees and hands, whereas this route of NSAID administration is not recommended in the therapy of pain associated with hip osteoarthritis, which is related to the poorer drug penetration into deeply located joints [21,49,50].

### 6.1. Pharmacodynamic Properties of Topical NSAIDs

The pharmacological rationale for topical NSAIDs relies on the localized, reversible inhibition of cyclooxygenase isoenzymes (primarily the inducible form, COX-2), which attenuates prostaglandin synthesis directly at the site of inflammation, conferring both anti-inflammatory and analgesic effects [51,52]. The clinical efficacy of topically applied formulations—predominantly diclofenac, ketoprofen, ibuprofen, and piroxicam—has been evaluated extensively [53,54], revealing distinct efficacy profiles depending on the acuity of the condition.

In the context of acute musculoskeletal pain, topical NSAIDs exhibit robust efficacy. For instance, diclofenac gel demonstrates an exceptionally low Number Needed to Treat (NNT)—defined in the pain literature as the number of patients required to be treated to achieve a predefined level of clinically meaningful pain relief (typically a ≥50% reduction in pain intensity) in one additional patient compared to placebo—of 1.8 [55]. Similarly high efficacy is observed for topical ketoprofen (NNT = 2.5) and piroxicam (NNT = 4.4) in acute settings [55].

However, the application of topical NSAIDs in chronic pain conditions (such as knee osteoarthritis) necessitates a more critical EBM appraisal. Although widely recommended by guidelines, their absolute clinical superiority over placebo in chronic settings is modest, largely due to a substantial vehicle-associated placebo response. In a pooled analysis of 2343 patients treated over a period of 6 to 12 weeks, topical diclofenac and ketoprofen achieved at least a 50% reduction in pain intensity in approximately 60% and 63% of patients, respectively [56]. These results must be interpreted cautiously, as the placebo response rates were exceptionally high (50% and 48%, respectively). This phenomenon underscores the strong psychological, and potentially mechanical (e.g., tissue massage during application), effects inherent to topical therapies.

Consequently, the NNT to achieve clinically meaningful pain reduction for one additional patient in chronic osteoarthritis remains relatively high: 6.9 for ketoprofen and 9.8 for diclofenac [56]. These findings indicate that while topical NSAIDs offer a crucial systemic safety advantage—particularly valuable in older, multimorbid populations—the actual pharmacological benefit in chronic pain is incremental, and the prominent placebo effect must be factored into realistic clinical expectations

### 6.2. Pharmacokinetic Properties of Topical NSAIDs

The pharmacokinetic rationale for topical NSAIDs relies on their amphiphilic properties, which facilitate penetration across the stratum corneum and subsequent distribution into underlying tissues [27,57,58]. Upon application, these agents achieve therapeutic concentrations locally in the dermis, fascia, muscle, synovial membrane, and articular cartilage without necessitating high systemic exposure [59,60]. For instance, it has been demonstrated that topical application of 5% ibuprofen gel (16 g) yields higher local tissue concentrations in the underlying fascia and muscle compared to an 800 mg oral dose [61].

Critically, while synovial fluid concentrations often mirror those in the systemic circulation, the absolute plasma concentrations following topical administration typically remain below 5% to 10% of the values observed after equivalent oral dosing [62,63]. Furthermore, specific agents demonstrate pronounced targeted tissue affinity; topical diclofenac, for example, achieves concentrations in the synovial membrane that are 10 to 20 times higher than those in either the synovial fluid or plasma [64]. This highly localized tissue accumulation, which is sustained across multiple applications [65], underpins the profound “systemic-sparing” safety advantage of topical NSAIDs.

Beyond their firmly established role in nociceptive and inflammatory musculoskeletal pain, topical NSAIDs have been hypothesized to offer therapeutic benefits in neuropathic pain conditions. This hypothesis is predicated on the pathophysiological involvement of localized inflammatory mediators in peripheral nerve sensitization [66,67]. However, from a rigorous EBM standpoint, this mechanistic rationale must not be conflated with proven clinical efficacy. While isolated, small-scale clinical trials and observational data suggest that topical diclofenac (1.5–5%) might transiently reduce pain intensity in conditions such as postherpetic neuralgia, complex regional pain syndrome (CRPS), and craniofacial neuropathies [68], these findings remain exploratory. They lack confirmation from large, double-blind, vehicle-controlled randomized trials. Consequently, topical NSAIDs are not currently recognized by major clinical guidelines as standard treatments for neuropathic pain. Similarly, while ketoprofen is occasionally incorporated into off-label compounded topical formulations for neuropathic pain [20,69,70], such polypharmacy practices remain highly investigational and are driven more by theoretical synergy than by robust clinical evidence (Table 3).

## 7. Local Anesthetics

The analgesic effect of local anesthetics is a frequently utilized component of topical analgesia. Most commonly, topical preparations contain lidocaine, tetracaine, or prilocaine [19]. These drugs, acting as local analgesics, stabilize the neuronal cell membrane and inhibit the opening of voltage-gated sodium channels, which consequently results in the disruption of sensory stimulus conduction [75,76]. By interacting with keratinocytes, endothelial cells, leukocytes, and mast cells, they also inhibit the release of inflammatory mediators [77]. Lidocaine is available on the market in various pharmaceutical dosage forms, i.e., gels, creams, patches, or aerosols, in different concentrations. Topically applied 5% lidocaine patches are one of the most well-established elements of topical therapy for focal neuropathic pain, particularly in postherpetic neuralgia [78,79]. Furthermore, a reduction in pain symptoms has been observed in postoperative neuropathy, diabetic polyneuropathy, chemotherapy-induced neuropathy, and in patients with predominant nociceptive pain in the course of carpal tunnel syndrome [80,81]. Long-term, topical application of lidocaine in the treatment of neuropathic pain provides sustained pain reduction, with a concurrent absence of systemic adverse effects [82]. The diverse pharmacokinetic properties of local anesthetics allow for the combination of several drugs. For example, an increased analgesic effect can be achieved by combining lidocaine with 7% tetracaine [83] or 2.5% prilocaine [84]. Such preparations are typically used in the treatment of acute pain. A topically applied drug containing lidocaine and prilocaine reduces pain symptoms arising from cuts and lacerations, and also effectively relieves pain caused by venous leg ulcers [84,85]. While the 5% lidocaine patch is a cornerstone of topical neuropathic pain management—largely due to its minimal systemic absorption (approximately 6%) and a highly favorable safety profile restricted primarily to mild local skin reactions [86]—the methodological robustness of the supporting evidence varies significantly across clinical indications. For instance, in localized diabetic peripheral polyneuropathy, a comparative trial involving 204 patients suggested that the lidocaine patch was as effective as pregabalin, with significantly better tolerability [87]. However, the short 4-week duration of this treatment limits the ability to extrapolate these findings to the long-term management of chronic neuropathic states.

Similarly, reports indicating high efficacy rates in conditions such as complex regional pain syndrome (up to 75% pain relief) [88] or postherpetic neuralgia (67% early response) [89,90] must be interpreted with methodological caution. Many of these findings are derived from open-label designs, observational cohorts, or highly selected patient phenotypes, making them highly susceptible to selection bias and the placebo effect. Furthermore, the use of topical local anesthetics in non-specific or predominantly nociceptive conditions, such as lower back pain [91], lacks high-quality evidence and remains a controversial, off-label practice. Consequently, while lidocaine patches represent an excellent, evidence-based option for focal neuropathic pain—especially in patients unable to tolerate the systemic side effects of gabapentinoids—their broader application is often overstated in the literature and still requires confirmation through rigorous, long-term randomized controlled trials.

### Capsaicinoids

Capsaicin is a natural plant-derived alkaloid found in the fruits of the Capsicum genus, which has found application in pain management, especially neuropathic pain, in the form of topical preparations. The action of capsaicinoids, which include capsaicin, involves binding to the transient receptor potential vanilloid 1 (TRPV1) receptor, which subsequently activates a non-selective cation channel on nociceptors [92,93]. Initial activation causes the release of various neuropeptides, but repeated topical application or a single exposure to high concentrations of capsaicin leads to overstimulation and, consequently, TRPV1 desensitization, depletion of neuropeptides, and reversible degeneration of epidermal nerve fibers [93]. TRPV1 is a Ca^2+^-selective member of the family of transient release potential ion channels, which sense heat. TRPV1 in the prelimbic and infralimbic cortex has also been revealed to mediate neuropathic pain. TRPV1 is broadly distributed in tissues of the brain, bladder, kidneys, intestines, epidermal keratinocytes, glial cells, liver, polymorphonuclear granulocytes, mast cells and macrophages. Capsaicin is an agonist of TRPV1 that reduces its activation threshold. Uniquely, after TRPV1 has been activated by capsaicin, the receptor enters a long-lasting refractory state, in which it does not respond to mechanical pressure, pain or inflammatory agents. This so-called ‘defunctionalization’ results from the closing of the channel pore due to conformational changes that depend on extracellular Ca^2+^. To what extent this transient ‘defunctionalization’ explains the observed analgesic effects of capsaicin remains unclear [94]. The analgesic effect of capsaicin is dose-dependent and can persist for several weeks; this applies particularly to high-concentration (8%) topical preparations, which are used as a single application [95]. Over-the-counter topical preparations, i.e., creams, gels, and patches containing capsaicin in lower concentrations (0.025–0.075%), require systematic and repeated application [96]. Due to its local mechanism of action and minimal systemic absorption, capsaicin exhibits a favorable safety profile, and its adverse effects are primarily limited to transient local reactions such as burning, erythema, edema, and pruritus, which occur more frequently with the use of high concentrations. Hence, the application of an 8% patch should be carried out adhering to appropriate procedures under the supervision of medical personnel [93]. Adverse effects can be mitigated by the prior application of topical local anesthetics or by cooling the site after patch application [93]. Since 2025, 0.075% capsaicin has been classified as a second-line drug in the therapy of peripheral neuropathic pain [17]. The therapeutic efficacy of capsaicin in the form of patches containing higher capsaicin concentrations (8%) has been confirmed in the treatment of various forms of neuropathic pain: chemotherapy-induced peripheral neuropathy, HIV-associated neuropathy, neuropathic back pain, painful diabetic neuropathy, and postherpetic neuralgia [97]. The clinical utility of the high-concentration (8%, 179 mg) capsaicin patch has recently been evaluated in real-world settings, such as the 2024 prospective QUCIP study involving breast cancer survivors with chemotherapy-induced peripheral neuropathy (CIPN). While such observational data suggest potential benefits in daily practice, they must be critically appraised against significant methodological limitations. Primarily, the observational design fails to control for the placebo effect—a particularly critical confounding factor in capsaicin research. The intense local reactions (burning, erythema) characteristic of high-concentration capsaicin virtually unblind patients, acting as a powerful active placebo that can inflate perceived efficacy.

Acknowledging these methodological caveats and the heterogeneity of clinical responses, the 2025 update to the NeuPSIG guidelines prudently positions the 8% capsaicin patch strictly as a second-line option for localized peripheral neuropathic pain [98]. This classification accurately reflects a realistic consensus: while the patch offers high predictability of systemic safety and avoids polypharmacy-related drug interactions, its average efficacy remains moderate. Furthermore, its clinical positioning is intrinsically limited by the requirement for supervised in-office application, potential need for pre-medication due to application-site pain, and high therapy costs

## 8. Topical Co-Analgesics: Menthol and Camphor—Significance in Pain Management

In pharmacy practice, a vast number of topical preparations recommended “for pain” are combinations based on cooling/warming substances, primarily containing menthol and camphor [99].

### 8.1. Menthol

Menthol, a natural terpene compound typically derived from peppermint oil (*Mentha piperita*), acts primarily through the modulation of cutaneous sensory receptors, specifically the TRPM8 (transient receptor potential melastatin 8) cold-sensing ion channel [100]. Activation of TRPM8 elicits a rapid cooling sensation, which, alongside the modulation of voltage-gated sodium channels [101,102,103], attenuates nociceptive signal conduction. Furthermore, menthol utilizes the phenomenon of counter-irritation, where the non-painful thermal stimulus competitively inhibits pain perception at the spinal level [101,104,105].

However, from an evidence-based medicine (EBM) perspective, this exact mechanism of action presents a fundamental methodological challenge. The intense sensory feedback (cooling and tingling) produced by menthol (typically used in 1% to 10% concentrations) virtually eliminates the possibility of effective blinding in clinical trials. Consequently, menthol inherently acts as a powerful active placebo, making it exceedingly difficult to untangle its true pharmacological efficacy from robust placebo responses in clinical settings.

Due to the lack of high-quality, rigorously blinded, and long-term randomized controlled trials, the clinical role of topical menthol must be interpreted with caution. While its minimal systemic absorption ensures a highly favorable safety profile (limited mostly to mild, transient application-site erythema or burning) [101,106], its utility is strictly confined to the short-term, temporary symptomatic relief of mild-to-moderate acute musculoskeletal or post-traumatic pain [107,108]. Menthol should be regarded as a subjective symptom-reliever and a transient adjunct, rather than a primary, evidence-supported therapy for chronic pain management [109].

### 8.2. Camphor

A compound found in certain trees of the laurel family (Lauraceae); it was traditionally extracted primarily from the bark, twigs, and wood of the camphor tree or produced synthetically. Camphor in topical preparations is typically present alongside other substances with anti-inflammatory and analgesic properties, acting as an adjuvant/co-analgesic (supporting anti-inflammatory and analgesic properties) [110]. It activates the TRPV1 receptor—a channel sensitive to heat/pain (the same one that responds to capsaicin from chili peppers)—and causes its initial stimulation, followed by desensitization (decreased reactivity), which can suppress pain conduction. This constitutes one of the molecular foundations of its analgesic action when applied to the skin [111]. It also activates the TRPV3 thermal receptor, a receptor associated with the sensation of warmth and the modulation of pain. Such modulation of sensory receptors leads to a decrease in pain signaling within the skin and subcutaneous tissues. Camphor induces localized skin hyperemia, which produces a “warming” sensation, improves blood flow, and may aid in the clearance of inflammatory mediators [112].

However, the rigorous EBM assessment of topical camphor reveals severe methodological limitations. Much like menthol, camphor relies on the principle of counter-irritation. The pronounced localized warming sensation and visible skin hyperemia it produces completely compromise patient and investigator blinding in clinical trials. Consequently, isolating its true pharmacological efficacy from a robust active-placebo response is nearly impossible. Given the profound lack of high-quality, standalone, randomized controlled trials, camphor cannot be endorsed as a primary analgesic agent. Its clinical role remains strictly limited to that of a short-acting, symptomatic counter-irritant used adjunctively for temporary relief of mild musculoskeletal complaints (Table 4).

## 9. Topical Therapy in Neuropathic Pain (Ketamine, Phenytoin, Clonidine, Amitriptyline, Gabapentin, Doxepin)

The efficacy of pharmacotherapy regarding neuropathic pain remains very limited. The 2025 NeuPSIG systematic update highlights the “modest efficacy” of many therapies, which is associated with, among other factors, the heterogeneity of mechanisms and patient phenotypes in trials [116]. The mechanism of action is known: applied to the skin, it blocks hyperexcitable receptors and ion channels directly in the damaged tissue. Clinical observations are very promising; unfortunately, their number is quite low [117]. Technological difficulties also arise. Preparing a stable formulation that effectively crosses the epidermal barrier and reaches nerve endings requires advanced pharmaceutical vehicles and high precision. Topical therapy unfortunately still requires refinement [118].

### 9.1. Doxepin

Doxepin belongs to the group of tricyclic antidepressants with strong antihistamine activity; it has a proven antipruritic effect [119]. In the form of a topical preparation, the drug was used in patients with chronic neuropathic pain encompassing symptoms such as: shooting pain, burning, numbness, paresthesia, and hypersensitivity (allodynia). The use of topical tricyclic antidepressants with antihistamine properties, such as doxepin, either alone or combined with low-dose capsaicin, represents an exploratory approach rather than an established clinical standard. While one trial involving 200 patients allocated to 3.3% doxepin, 0.025% capsaicin, a combination of both, or placebo suggested that the combined formulation provided the most rapid onset of analgesia [120], a critical appraisal of these findings reveals significant methodological caveats.

Most notably, the study reported a statistically significant reduction in pain intensity across *all* study arms, including the placebo group. This robust vehicle-associated placebo response strongly confounds the interpretation of the true pharmacological efficacy of the active ingredients. Furthermore, with only 50 patients per treatment arm, the trial is statistically underpowered to definitively establish synergistic effects or long-term outcomes. Consequently, while the combination of doxepin and capsaicin may offer a theoretical mechanistic rationale, the current evidence is strictly hypothesis-generating. Compounded doxepin creams should not be recommended in routine practice and remain an experimental option only when standard, evidence-based topical therapies have been exhausted.

### 9.2. Amitryptyline and Ketamine

Despite plausible theoretical mechanisms—such as the peripheral blockade of NMDA receptors by ketamine and the multidirectional action of amitriptyline on sodium channels and nociceptors [117,121,122,123,124]—the translation of these pharmacodynamic concepts into clinical efficacy has largely failed. Consequently, their routine use in compounded topical formulations for neuropathic pain remains clinically unjustified.

Although pharmacokinetic studies indicate that topical ketamine (typically 1% to 5%) and amitriptyline (2% to 4%) achieve negligible systemic absorption [125,126,127], this favorable safety profile cannot compensate for the profound lack of high-quality efficacy data. Most randomized controlled trials (RCTs) have consistently failed to demonstrate a statistically significant superiority of these creams over placebo [17]. The existing literature is heavily compromised by critical methodological limitations, including small sample sizes, high risk of bias, and unblinding issues.

Furthermore, a major confounding factor in evaluating these agents is the extreme heterogeneity of the pharmaceutical vehicles used (ranging from traditional ointments to advanced liposomal bases) [125,127,128,129]. This lack of standardization significantly alters epidermal penetration, rendering cross-study comparisons virtually impossible and making it difficult to distinguish true pharmacological analgesia from robust vehicle-associated placebo responses.

Therefore, while isolated reports suggest potential benefits in highly specific subgroups (e.g., radiation-induced neuropathy or severe allodynia), these observations must be interpreted as strictly hypothesis-generating. Until standardized, multicenter, and rigorously blinded RCTs are conducted, topical amitriptyline and ketamine cannot be recommended in any standard neuropathic pain treatment algorithms. They must be regarded exclusively as experimental options [17,130,131].

### 9.3. Phenytoin

Topical phenytoin has been proposed as a peripheral sodium channel blocker aimed at reducing nerve hyperexcitability [132]. While preliminary pharmacokinetic studies suggest that creams containing up to 20% phenytoin result in negligible systemic absorption and undetectable plasma levels [133], this theoretical safety advantage cannot be conflated with clinical efficacy. The current body of evidence supporting its use in neuropathic pain is distinctly preliminary, highly heterogeneous, and limited in scope.

Recent literature has introduced novel trial designs, such as the EPHENE project for chronic idiopathic axonal polyneuropathy (CIAP), which utilizes a ‘DOBRET’ (double-blind response test) to prospectively identify potential ‘responders’ before initiating long-term therapy. While such enriched enrollment strategies attempt to mitigate the high failure rates and placebo responses typical in topical pain trials, they concurrently highlight a critical limitation: topical phenytoin lacks broad, generalized efficacy across the patient population.

Consequently, preliminary reports suggesting that an early treatment response might predict long-term pain reduction must be interpreted strictly as hypothesis-generating observations [134]. Without definitive, peer-reviewed data from fully powered, large-scale randomized controlled trials, the clinical utility of topical phenytoin remains unestablished. At present, it must be classified as an entirely experimental intervention and cannot be endorsed as an evidence-supported clinical recommendation

### 9.4. Topical Gabapentin

While systemic gabapentin is a well-established treatment for neuropathic pain conditions, the clinical utility of topical gabapentin remains entirely theoretical. The pharmacodynamic rationale posits that topically applied gabapentin could block peripherally overexpressed calcium channels in chronic pain states, thereby inhibiting glutamate and substance P release without inducing central nervous system side effects [117,135,136,137,138,139].

However, the current literature evaluating topical gabapentin formulations is overwhelmingly restricted to preclinical in vivo models [140,141]. Although topically applied gels have demonstrated anti-allodynic effects in rodent models of diabetic neuropathy and vulvodynia, extrapolating these animal findings directly to human clinical efficacy is a significant methodological overreach. Assertions in the literature that topical gabapentin exhibits ‘comparable activity profiles’ to systemic administration while avoiding systemic toxicity [136,141,142,143] are premature and lack validation from robust human data.

The translation from animal models to human pathophysiology is heavily confounded by variables such as human epidermal barrier differences, formulation-dependent absorption limits, and the profound vehicle-associated placebo responses typical of human topical pain trials. Consequently, suggesting that topical gabapentin constitutes a ‘better alternative’ for clinical neuropathic pain management is currently unsupported by evidence-based medicine. At present, topical gabapentin is not a clinical recommendation but strictly a preclinical hypothesis that requires comprehensive evaluation through large, randomized, double-blind, vehicle-controlled human trials (Table 5).

## 10. How to Understand Combination Therapy in Topical Analgesia

The principle of combining drugs with different mechanisms of action in order to increase the analgesic effect and limit adverse events is well-known in chronic pain management [155]. In the context of topical treatment, the key benefit is the ability to reduce the dose or exposure time to systemic drugs (e.g., oral NSAIDs), and thus reduce the risk of adverse events (gastrointestinal, cardiovascular, and renal) [156]. Osteoarthritis (OA) guidelines are very specific in this regard: “prefer local therapies with the lowest systemic exposure before resorting to oral NSAIDs”—this can be observed in the recommendations of the ACR/Arthritis Foundation and OARSI, as well as in AAOS documents [21,49,156,157]. Pain is a subjective experience, so its proper assessment is very important in both clinical practice and research. The most commonly used tools for measuring pain intensity are the Numeric Rating Scale (NRS) and the Visual Analog Scale (VAS). The NRS asks patients to rate their pain on a scale from 0 to 10, where 0 means no pain and 10 means the worst pain imaginable. Because it is simple, easy to use, and reliable, it is one of the most widely used pain assessment tools in everyday clinical practice. The VAS requires patients to mark their pain intensity on a 100 mm line, with one end representing no pain and the other representing the worst pain imaginable. It is sensitive to changes in pain intensity but may be more difficult to use in older adults and in patients with cognitive impairment or limited manual abilities [158].

Other tools, such as the Verbal Rating Scale (VRS), which uses descriptive words to rate pain intensity, and the Brief Pain Inventory (BPI), which also evaluates how pain affects daily activities, are sometimes used as well. The choice of pain scale depends on the purpose of the assessment and the characteristics of the patient, but NRS and VAS remain the most commonly used and best-validated tools for pain assessment [159].

Pain assessment scales differ in their advantages and limitations. The Numeric Rating Scale (NRS) is simple, quick, and easy to use, making it one of the most common tools in clinical practice. However, it requires patients to understand numerical concepts. The Visual Analog Scale (VAS) is more sensitive to small changes in pain intensity but may be more difficult to use in older adults and patients with cognitive or manual impairments. The Verbal Rating Scale (VRS) is easy to understand and useful in some patient groups, although it is less precise. The Brief Pain Inventory (BPI) provides additional information on how pain affects daily functioning but requires more time to complete [158,159].

The choice of a pain assessment tool depends on the purpose of the evaluation, the clinical setting, and patient characteristics, including age, cognitive status, communication skills, and ability to complete the scale. In practice, physicians select the tool that is most appropriate and feasible for a given patient.

### A Simple Practical Algorithm

In practice, the selection of topical therapy can be based on three fundamental questions:

- Is the pain localized and “accessible” to topical pharmacotherapy? (knee—yes, hip—often no). A thoroughly obtained medical history is essential.
- Does the mechanism suggest an “inflammatory/nociceptive” or “neuropathic” origin? (because this alters the choice of the applied drug: NSAIDs vs. lidocaine/capsaicin).
- Is the patient at high risk for adverse events from systemic therapies? (age, kidneys, heart, gastrointestinal tract, polypharmacy) [17,21,49,55].

## 11. Summary

Pain remains a ubiquitous clinical challenge, and the chronification of pain represents an immense global health burden. To optimize outcomes, treatment cannot rely on habitual prescribing; it must be strictly aligned with the underlying pathophysiological mechanism (nociceptive, neuropathic, or nociplastic) and involve a critically appraised choice of both the agent and its route of administration. Within the landscape of topical analgesia, three interventions currently possess the most robust evidence base, though each requires mindful clinical application and an understanding of its methodological limitations:

Topical NSAIDs: Strongly supported by current guidelines as a first-line therapy for localized osteoarthritis (particularly of the knee and hand) and acute musculoskeletal injuries. While their primary clinical value lies in minimizing systemic exposure and avoiding gastrointestinal/cardiovascular toxicity, their absolute analgesic superiority over placebo is often modest, largely due to prominent vehicle-associated placebo responses.

5% Lidocaine Patch: A well-established option for focal neuropathic pain (most notably postherpetic neuralgia), characterized by minimal systemic absorption and an excellent safety profile. However, extrapolating its use to off-label indications or broad, non-specific pain syndromes lacks rigorous evidence and is not currently justified by high-quality randomized trials.

8% Capsaicin (179 mg) Patch: Positioned primarily as a second-line option for localized peripheral neuropathies (e.g., PHN, PDPN). While clinical trials and real-world data support its utility, its evidence base is inherently confounded by trial unblinding (due to intense application-site reactions), and its routine use is limited by high costs and the necessity for supervised, in-office administration.

Ultimately, while topical therapies offer a crucial safety advantage in multimodal pain management, maintaining a critical, evidence-based perspective is essential to distinguish proven pharmacological benefits from placebo effects and methodological overreach.

## 12. Results

Current evidence supports the integration of specific topical analgesics—namely NSAIDs, 5% lidocaine, and 8% capsaicin patches—into evidence-based pain management algorithms, provided they are used for defined, localized conditions. In stark contrast, the clinical application of newer compounded therapies (including topical ketamine, amitriptyline, phenytoin, gabapentin, clonidine, and doxepin) remains fundamentally unsupported by robust evidence-based medicine (EBM) standards.

The existing literature regarding these experimental agents is severely compromised by pervasive methodological flaws. The vast majority of trials are significantly underpowered, feature inadequate blinding, and fail to consistently demonstrate statistical superiority over placebo. This lack of efficacy is largely attributable to the profound vehicle-associated placebo response inherent to topical applications, which frequently masks the true (or absent) pharmacological effect of the active ingredients.

Furthermore, synthesizing this data into clinical recommendations is precluded by extreme inter-study heterogeneity. Trials differ drastically in patient phenotyping, pain etiologies, treatment durations, and—crucially—formulation pharmacology (e.g., varying drug concentrations and unstandardized delivery vehicles). The validity of the available data is additionally threatened by a high risk of publication bias, where positive, small-scale, or open-label studies are disproportionately overrepresented in the literature. Moreover, the stringent exclusion criteria typical of these preliminary trials severely limit the external validity and generalizability of their findings to everyday, multimorbid clinical populations.

Consequently, labeling these advanced compounded therapies as ‘promising’ constitutes a methodological overstatement. Until definitive, fully powered, long-term, and rigorously vehicle-controlled randomized trials are conducted, the routine use of these newer topical approaches remains unjustified. They must be strictly classified as investigational, hypothesis-generating tools rather than established therapeutic strategies

## 13. Conclusions

Topical analgesics offer a vital alternative to systemic pharmacotherapy by achieving localized pain relief while significantly mitigating systemic toxicity. Robust evidence-based medicine (EBM) unequivocally supports the targeted use of topical NSAIDs, 5% lidocaine, and high-concentration capsaicin for specific, localized pain phenotypes. However, while the theoretical concept of combining diverse active agents in compounded formulations is pharmacodynamically appealing, the clinical translation of this multimodal strategy remains largely unsubstantiated.

Consequently, any enthusiasm for emerging topical treatments—such as ketamine, phenytoin, or compounded gabapentinoids—must be strictly curtailed. The current evidence base for these agents is profoundly deficient, relying heavily on small, methodologically flawed trials that fail to adequately control for massive vehicle-associated placebo responses. Therefore, designating these experimental formulations as ‘promising’ is clinically misleading; they must be strictly classified as hypothesis-generating concepts rather than evidence-supported therapeutic options.

Future advancement in this field absolutely necessitates fully powered, double-blind, vehicle-controlled randomized clinical trials. Until such rigorous data establish unequivocal pharmacological superiority over placebo, these novel topical therapies must remain entirely outside the scope of routine clinical guidelines.

The effective and safe management of chronic pain, particularly neuropathic pain, continues to be a formidable clinical challenge. While the pursuit of optimized topical delivery systems is pharmacologically justified, clinical implementation must be driven exclusively by uncompromising, high-quality evidence rather than theoretical optimism or anecdotal success.

## Figures and Tables

**Table 1:** Pathophysiological and clinical characteristics of the three main types of pain.

Type of Pain	Definition	Pathophysiology	Example Clinical Phenotypes	Rationale for Topical Therapy
Nociceptive	Pain arising from activation of nociceptors in non-neural tissues	Prostaglandins, cytokines; “peripheral sensitization” of nociceptors	Pain with movement, tenderness, functional limitation; often an inflammatory component	Topical NSAIDs in injuries and OA; patches/gels when the problem is superficial and localized [21]
Neuropathic: Central Peripheral	Pain caused by a lesion or disease of the somatosensory nervous system	Sodium channels and ectopic discharges; NMDA-dependent plasticity; neuroinflammation and release of pro-inflammatory mediators by glial cells	Burning, shooting, allodynia; “focal” or dermatomal area	5% lidocaine in focal pain; 8% capsaicin in focal pain [22]
Nociplastic	Pain arising from altered nociception despite the lack of clear evidence of actual or threatened tissue damage and the lack of disease of the somatosensory system	Central sensitization, dysregulation of pain inhibition, biopsychosocial factors	Diffuse pain, “excess” of symptoms relative to changes; comorbidity with mood and cognitive function disorders; pain is always chronic in nature	Topical medications rather as a supportive element when there is a local pain component; multimodal strategy is more important [23]

OA—osteoarthritis. NSAIDs—non-steroidal anti-inflammatory drugs.

**Table 2:** Characteristics of topical drug formulations.

Formulation	Maximization of Effect	Limitations	Typical Clinical Situations
Gel (hydrogel, emulgel)	rapid release, pleasant application, cooling effect; good patient acceptance	may dry faster; need for frequent application	soft tissue injuries, tendinopathies, superficial pain; knee/hand OA [34]
Cream/ointment	occlusion, hydration, contact time	sticky feeling; variable base-dependent penetration	“shallow” pain, dry areas, need for longer contact time; preparations with menthol/camphor [35]
Solution/spray	fast and even coverage; less “rubbing”	risk of uneven dosing; evaporation	situations where you do not want to massage (e.g., acute injury) [36]
Topical patch (NSAIDs, lidocaine, capsaicin)	sustained release, fewer “fluctuations”, convenience; potential for mechanical effect (skin protection)	limited surface area; risk of skin reactions; cost	focal pain/limited area: postherpetic neuralgia (lidocaine), focal neuropathic pain (capsaicin), OA (selected NSAID patches) [37]

OA—osteoarthritis. NSAIDs— non-steroidal anti-inflammatory drugs.

**Table 3:** Topical NSAIDs—comparison of substances and drug formulations.

Substance/Formulation	Best Indications (EBM)	Strength of Clinical Evidence	Adverse Effects
Diclofenak (gel/emugel, solution, patch)	acute soft tissue pain; knee/hand OA	very good data in acute pain: NNT~1.8 for selected emulgel formulation [55]; in OA moderate efficacy, some studies 6–12 weeks [56]	local irritation; systemic exposure 5–17× lower vs. oral diclofenac [71]
Ketoprofen (gel, patch)	acute soft tissue pain; OA (esp. knee)	in acute pain NNT~2.5 for gel [55]	important: risk of photoallergy/phototoxicity; sun protection recommendations [72]
Ibuprofen (gel)	acute musculoskeletal pain	good data in acute injuries	usually good tolerance; keep in mind skin irritations [73]
S-flurbiprofen patch (SFPP)	knee OA; selected perioperative indications	randomized and comparative trials vs. placebo/vs. diclofenac	dermatitis/local reactions; good choice when convenience and sustained release are required [74]

**Table 4:** Menthol and camphor–TRP mechanisms and practical clinical interpretation.

Substance	Main Receptor Targets	Effect Described by Patients	Most Common Applications	EBM Limitations
Menthol	TRPM8 (mainly), under certain conditions also TRPA1 [110]; possible coupling with pain inhibition systems [105]	cold, rapid symptomatic relief [107]	muscle, overuse, and post-traumatic pain; component of combination products	clinical data exist but are heterogeneous; there is no RCT base as consistent as for NSAIDs/lidocaine [113]
Camphor	TRPV1 (activation and desensitization) [114], TRPV3 [112], TRPM8; TRPA1 inhibition	sensation of warming/cooling depending on dose and context; pain modulation	warming preparations [115]	reviews indicate potential, but the number of robust clinical trials is limited [101]

**Table 5:** Topical medications in neuropathic pain: level of evidence, mechanisms, risks.

Substance/Preparation	Receptor/Molecular Mechanism	Clinical Data in Humans	Application	Adverse Effects
Lidocaine 5% patch	sodium channel (NaV) blockade and decreased excitability of sensory fibers	good data in postherpetic neuralgia and localized pain; low systemic exposure	focal pain, allodynia, patient at risk of systemic side effects	skin reactions; caution on damaged skin [78,87,144]
Capsaicin 8% patch	TRPV1 agonist → defunctionalization of nerve endings; effect lasting weeks	RCT in PHN [145]; RCT in PDPN [146]; comparison with pregabalin; real-world in CIPN (QUCIP) [147]	focal neuropathic pain, when the area can be covered by a patch	pain/burning during application, skin reactions; in-office procedure
Clonidine gel	α2 agonist; modulation of pain signaling in the skin; effect dependent on preserved nociceptors	RCT in PDN with an efficacy signal in a selected phenotype; Cochrane 2022: uncertainty of evidence [23,30,37,116]	PDN with preserved function of skin nociceptors	variable response; limited certainty of evidence on a population level—*need for large randomized trials*
Doxepin cream	multidirectional: TCA + antihistamine effect; possible peripheral effects	RCT: doxepin and doxepin + capsaicin reduced neuropathic pain [120]; reactions usually mild, lack of anticholinergic symptoms—which is important	selected neuropathic pain when other topical options are unavailable	sedation upon absorption, irritation [148]
Topical ketamine/ketamine + amitriptyline	NMDA antagonism; sodium and calcium channel blockade; effect on peripheral opioid receptors.	weak population data; lack of statistical superiority over placebo in general RCTs [130,149]. Results of the latest meta-analyses indicate low-quality evidence [150].	rather experimental or in research protocols; possibly in patients with a lack of other options and focal pain	lack of stable evidence [125];
Phenytoin cream (10–30%)	sodium channel blockade; modulation of nerve excitability; potential in neuropathic pain	growing data: observational protocols and papers on the lack of plasma levels—efficacy demonstrated [151]. Studies from 2025 confirm the correlation of rapid relief with long-term effect [134]	as a candidate for the “response test → treatment of responders” strategy. Best with patient phenotyping [132,133]	still a limited number of large RCTs [152];
Compounded creams (multiple ingredients)	sodium channels, α2, NMDA, etc.—trendy approach multimodal, multidirectional	research gap	research gap	research gap [153,154]

→ indicates “leads to”.

## Data Availability

No new data were created or analyzed in this study.
